# Bacteria in Extracorporeal Membrane Oxygenation Circuit Clots of a Patient With Persistent Bacteremia: A Case Report

**DOI:** 10.1097/MAT.0000000000001980

**Published:** 2023-05-17

**Authors:** Joppe G. Drop, Latisha Verhage, Mireille van Westreenen, Enno D. Wildschut, Matthijs de Hoog, Heleen van Beusekom, C. Heleen van Ommen

**Affiliations:** From the * Department of Pediatric Hematology, Erasmus MC Medical Center-Sophia Children’s, Rotterdam, The Netherlands; † Department of Neonatal and Pediatric Intensive Care, Division of Pediatric Intensive Care, Erasmus University Medical Center, Rotterdam, The Netherlands; ‡ Department of Cardiology, Erasmus MC University Medical Center, Rotterdam, The Netherlands; § Department of Medical Microbiology and Infectious Diseases, Erasmus MC University Medical Center, Rotterdam, the Netherlands.

**Keywords:** pediatrics, extracorporeal membrane oxygenation, bacteria, thrombosis, circuit change

## Abstract

A neonate with pulmonary hypertension was supported with extracorporeal membrane oxygenation (ECMO). During ECMO support, the patient developed *Enterococcus faecalis* bacteremia, treated with targeted antibiotics. Despite the maximum dose of antibiotics, routine blood cultures remained positive throughout the ECMO treatment. A circuit change was performed due to buildup of thrombotic material and disseminated intravascular coagulation (DIC) inside the circuit. Thrombus formation was more extensive in the first than the second circuit. Gram-positive diplococci were present in all initial circuit clots and gram-positive masses surrounded by fibrin were found inside thrombi of the second circuit. Scanning electron microscopy (SEM) revealed a dense fibrin network with embedded red blood cells and bacteria in the first circuit. In the second circuit, SEM analysis revealed scattered micro thrombi. Polymerase chain reaction for identification of bacteria in the thrombus of the first circuit showed the same bacteria as found in blood cultures and did not achieve a sufficient signal in the second circuit. This case report shows that bacteria can nestle in thrombi of an ECMO circuit and that there is a rationale for a circuit change in a patient with persistent positive blood cultures and DIC.

Extracorporeal membrane oxygenation (ECMO) is a supportive therapy for children with severe cardiac and/or pulmonary failure. Due to contact between blood and foreign material of the circuit and subsequent activation of the coagulation system, patients develop a prothrombotic state. To prevent thrombosis and maintain patency of the ECMO circuit, anticoagulation, usually unfractionated heparin (UFH), is used.

Despite high dosages of anticoagulants, thrombosis can develop inside ECMO circuits, leading to thromboembolism, hemolysis, and the need for a circuit change.^1^ According to the extracorporeal life support organization registry in 2020, circuit thrombosis developed in up to 35.5% and 25.8% of neonates and children on ECMO, respectively. In addition, a circuit change was reported in up to 9.5% of neonatal and 12.8% of pediatric ECMO patients.^2^

The etiology of clot formation inside pediatric ECMO circuits is not fully understood. An important factor appears to be the local hemodynamics, as most thrombi are found in locations with turbulent flow, that is, the connector junction sites (CJS), pump head, and oxygenators.^3,4^ Histological analysis of clots from different locations identified fibrin, platelets, red blood cells and von Willebrand factor in various amounts.^3,4^ The organization of thrombi inside ECMO circuits has been investigated in one study of 36 adult ECMO circuits. Scanning electron microscopy (SEM) showed deposits on gas exchange fibers with embedded platelets and red blood cells and large areas with pseudomembrane formation.^5^

Another contributing factor to clot formation might be the occurrence of infections. Infections contribute to a hypercoagulable state and are a known risk factor for thrombotic complications.^6^ However, the interaction between thrombus formation and composition and bacterial infections in ECMO patients is not known. In our hospital, blood stream infections in children undergoing ECMO are most frequently caused by coagulase-negative *Staphylococcal* spp, *Enterobacter* spp, and *Eschericia* spp (unpublished data). Despite aggressive antibiotic therapy, infections are therapy resistant in some patients. In the treatment of ongoing bacteremia, it is common clinical practice to change all foreign material inside the patient, including the ECMO circuit. However, there is still little scientific understanding of the benefits of a circuit change, while there are also risks involved. Therefore, we aim to study the rationale for a circuit change in ongoing sepsis. In this substudy, we describe the microscopic analysis of ECMO circuit thrombi of a neonate with a persistent bacteremia under the maximum dosages of antibiotics.

## Case

A term 3.2 kg neonate was started on veno-arterial ECMO 20 hours after birth, due to persistent pulmonary hypertension unresponsive to inotropics and vasopressants. On day 2 of ECMO support, the patient developed fever (37.5°C). On the same day, a routine blood culture was drawn and penicillin and gentamicin were empirically administered. In addition, the antibiotic regimen was switched to flucloxacillin and gentamicin due to worsening of the clinical situation. On day 3, the regimen was adapted to vancomycin, metronidazole, and cefotaxime, after finding Gram-positive cocci in the blood culture and due to suspicion of an abdominal infection focus. Gram-positive cocci proved to be *Enterococcus faecalis*, sensitive for amoxicillin, after which the antibiotic regimen was switched to the maximum dose of amoxicillin, cefotaxime, and metronidazole. Per protocol, daily blood cultures were performed, which remained positive throughout the whole antibacterial and ECMO treatment for *E. faecalis*. Coagulation tests revealed disseminated intravascular coagulation (DIC) with a platelet count of 6 × 10^9^/L, elevated d-dimers (11.5 mg/L) and C-reactive protein (59 mg/L). Therefore, platelet transfusions (target above 100 × 10^9^/L) and continuous plasma transfusions were initiated. The coagulation management with UFH was targeted on activated partial thromboplastin time between 85 and 120 seconds, and antifactor-Xa levels between 0.5 and 1.0 IU/mL. The heparin dose increased from 35 IU/kg/h after ECMO initiation to 50 IU/kg/h on day 6 of ECMO support. Due to excessive thrombus formation in the ECMO circuit in concurrence with DIC and positive blood cultures, a circuit change was performed. After the circuit change, hemodynamics slightly improved, but remained suboptimal with need for vasopressants, continuous plasma infusions and hemofiltration. On day 7, the patient died of hemodynamic and hemostatic complications. Postmortem examination was not performed.

## Methods and Results

**Figure 1.**
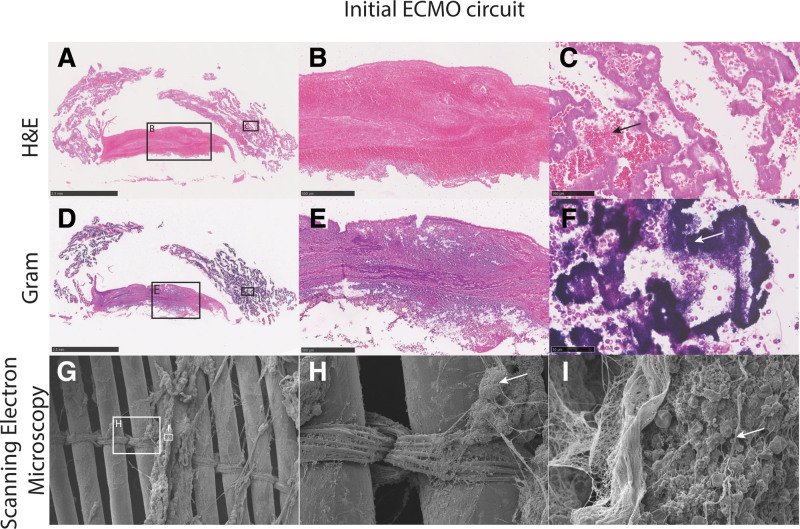
Representative images of the initial extracorporeal membrane oxygenation as overviews and details (indicated in overview images by boxes). Overview (**A**) and details (**B**: 20×; **C**: 40×) at the post oxygenator connector junction site, stained by Hematoxylin and Eosin, shows the presence of ample fibrin and platelets as well as some erythrocytes (**C**, arrow); At the same location, an overview (**D**) and details (**E**: 20×; F: 40×) are given, but stained with a Gram stain, showing the presence of bacteria (**F**, arrow); Scanning electron microscopy of oxygenator fibers and connecting structures (**G**: 30×; **H**: 150×; **I**: 1500×) shows the presence of groups of bacteria covered by fibrin strands (arrows in **H** and **I**).

**Figure 2.**
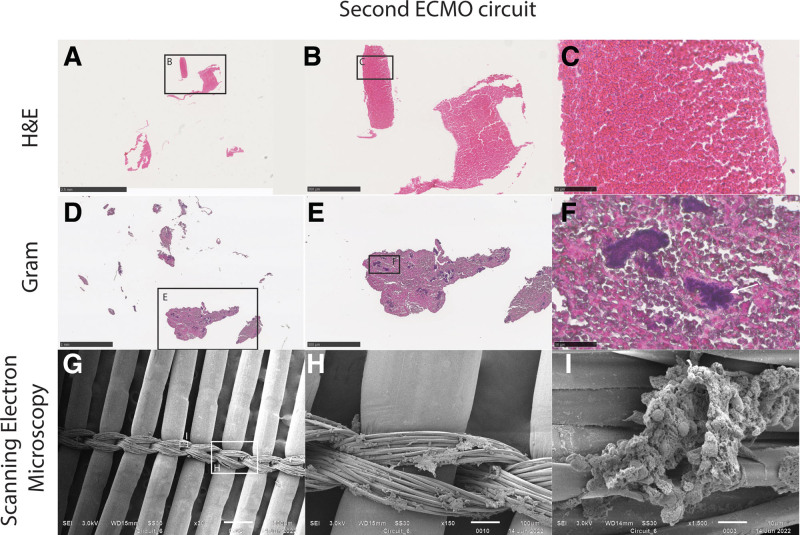
Representative images of the initial extracorporeal membrane oxygenation circuit. (**A**) Hematoxylin and Eosin Stain of post oxygenator connector junction site (10×); (**B**) Hematoxylin and Eosin Stain detail (20×); (**C**) Hematoxylin and Eosin Stain detail (40×); (**D**) Gram stain of post oxygenator connector junction site (10×); (**E**) Gram stain of post oxygenator connector junction site detail (20×); (**F**) Gram stain of post oxygenator connector junction site detail (40×); (**G**) Scanning electron microscopy of oxygenator fibers and connecting structures (30×); (**H**) Scanning electron microscopy of oxygenator fibers and connecting structures (150×); (**I**) Scanning electron microscopy of oxygenator fibers and connecting structures (1500×).

This is a substudy of the CHECKID study, a prospective observational study in pediatric ECMO patients (MEC-2018-0085). Two ECMO circuits were collected from one patient. The first circuit was obtained on the third day of ECMO support and the second circuit after the patient died, 7 days after ECMO initiation. The methods of sample collection and processing are described in Supplemental Digital Content 1, http://links.lww.com/ASAIO/B41. Macroscopically, clots were observed at all CJS and in the oxygenators of both ECMO circuits. In general, macroscopic thrombus formation was more extensive in the first circuit. While thrombi were adherent to the surface in the first circuit, they were freely floating in the lumen of the second circuit. Figures 1 and 2 show representative images of the histological and SEM analyses of both ECMO circuits. A layered pattern was observed in thrombi from CJS of the first circuit (Figure 1, B and D). This pattern was not observed in thrombi from the second circuit, in which thrombi had a uniform structure (Figure 2A–C). Gram stains showed the presence of Gram-positive diplococci in all thrombi of the initial circuit. Thrombi from the second circuit revealed Gram-positive aggregates surrounded by fibrin, embedded inside thrombi (Figure 2, D–F). A dense fibrin network with embedded red blood cells and bacteria was found on SEM analysis of the first circuit (Figure 1 G–I). In the second circuit, SEM analysis revealed scattered micro thrombi in the oxygenator (Figure 2, G and I). Polychain reaction (PCR) (16S rRNA PCR) of thrombus material of the first circuit showed the same bacteria as were found in the blood cultures (*E. faecalis*). PCR of the second circuit did not achieve a sufficient signal.

## Discussion

This substudy describes a neonate with ongoing bacteremia, despite aggressive antibacterial therapy, during ECMO support. The initial circuit contained more and differently organized thrombotic depositions and had a positive PCR, whereas the PCR was negative in the second circuit. In addition, the same bacteria were found in thrombi and blood cultures. These results suggest that changing the ECMO circuit reduces the thrombotic and bacterial load in the ECMO circuit in a patient with positive blood cultures and DIC.

Similar to other studies, the majority of circuit thrombi were found at locations with turbulent flow, including the CJSs and oxygenators of both circuits. These findings support the theory of Hastings *et al*. about the role of local hemodynamics in the development of thrombi in ECMO circuits.^3^ The mechanism behind the bacterial presence inside these thrombi remains unclear. We hypothesize that free floating bacteria from blood nestle in thrombi due to turbulent blood flow. Inside thrombi, bacteria divide and form bacterial masses (Figure 1F). The local inflammatory response, aggravated by bacteria, causes local activation of the coagulation system, leading to a dense fibrin network (Figure 1, G and I). Consequently, antibiotic treatment cannot reach these bacteria and is therefore less effective. Moreover, bacterial aggregates from these thrombi may disseminate throughout the ECMO circuit and the patient, creating new infection focus locations. When the ECMO circuit is changed, the canulas in the patient are not changed, as this would interrupt ECMO support. Yeo et al. described a significant association between ECMO canulas infection and an infection during ECMO support.^7^ Thus, it is likely that the canulas were also infected. In this substudy, the ECMO canulas were not analyzed.

Nonetheless, we suspect that bacterial aggregates in the blood originate from both canulas thrombi and infected thrombi in the circulation. It is likely that these aggregates ended up in thrombi of the second circuit, explaining our findings of Figure 2, D–F.

Although an ECMO circuit cannot be completely changed, our results suggest that a circuit change was partly effective in reducing the bacterial load, as the PCR signal of the second circuit did not achieve a sufficient signal. Unfortunately, blood cultures remained positive for *E. faecalis* 3 days after the circuit change, despite the high dose of antibacterial treatment. This might be explained by the interaction between antibiotics and the ECMO circuit that complicates dose optimization of antibiotics. The interplay between bacteria and thrombi in the ECMO circuit in this case highlights the importance of dose optimization of antibiotics.^8^

## Conclusions

This case report is the first to describe that bacteria can nestle in thrombi of the ECMO circuit. Furthermore, it suggests that there is a rationale for a circuit change in a patient with persistent positive cultures and DIC leading to a reduced bacterial and thrombotic load in the circuit. Finally, our findings underline the importance of prevention of thrombi in the ECMO circuit and optimization of antibacterial therapy in this group of patients.

## Supplementary Material



## References

[1] Dalton HJ Reeder R Garcia-Filion P; Eunice Kennedy Shriver National Institute of Child Health and Human Development Collaborative Pediatric Critical Care Research Network: Factors associated with bleeding and thrombosis in children receiving extracorporeal membrane oxygenation. Am J Respir Crit Care Med. 196: 762–771, 2017. 28328243 10.1164/rccm.201609-1945OC PMC5620676

[2] The Extracorporeal Life Support Organization. ECLS Registry Report, International Summary. (2020). Ann Arbor: Extracorporeal Life Support Organization, pp.1–39.

[3] Hastings SM Ku DN Wagoner S Maher KO Deshpande S: Sources of circuit thrombosis in pediatric extracorporeal membrane oxygenation. ASAIO J. 63: 86–92, 2017. 27660905 10.1097/MAT.0000000000000444

[4] Staessens S Moussa MD Pierache A: Thrombus formation during ECMO: Insights from a detailed histological analysis of thrombus composition. J Thromb Haemost. 20: 2058–2069, 2022. 35703468 10.1111/jth.15784 PMC9349827

[5] Lehle K Philipp A Gleich O: Efficiency in extracorporeal membrane oxygenation-cellular deposits on polymethylpentene membranes increase resistance to blood flow and reduce gas exchange capacity. ASAIO J. 54: 612–617, 2008. 19033775 10.1097/MAT.0b013e318186a807

[6] Drop JGF Wildschut ED Gunput STG de Hoog M van Ommen CH: Challenges in maintaining the hemostatic balance in children undergoing extracorporeal membrane oxygenation: A systematic literature review. Front Pediatr. 2020: 67, 6124. 10.3389/fped.2020.612467 PMC7772234 33392120

[7] Yeo HJ Yoon SH Lee SE: Bacterial biofilms on extracorporeal membrane oxygenation catheters. ASAIO J. 64: e48–e54, 2018. 29356690 10.1097/MAT.0000000000000750

[8] Gomez F Veita J Laudanski K. Antibiotics and ECMO in the Adult Population-Persistent Challenges and Practical Guides. Antibiotics (Basel). 11: 338, 2022. 10.3390/antibiotics11030338 PMC8944696 35326801

