# Supplementary material for: Bacteria in Extracorporeal Membrane Oxygenation Circuit Clots of a Patient With Persistent Bacteremia: A Case Report
Source: ASAIO J. 2023 May 17;69(11):e463–6. doi: 10.1097/MAT.0000000000001980 (PMC10602214; doi:10.1097/MAT.0000000000001980)
Supplement: Supplementary file 1 [file mat-69-e463-s001.pdf]

Supplement 1

Methods

After collection, the initial and second ECMO circuit were transported to the laboratory, where they were gently flushed with Ringer's lactate. After flushing, circuits were inspected for macroscopic clots. Thereafter, CJS before and after the pump and oxygenator and oxygenator fibers were collected using a sonic cutter (NE-80, NSK Nakanishi INC. Japan). After sampling, samples were cut into two pieces and fixed in buffered 4%paraformaldehyde-2%glutaraldehyde for 48 hours and stored in 0.1M cacodylate until further processing. Per sample, one piece was used to obtain slides for microscopic analysis and one for Scanning Electron Microscopy (SEM). Samples were dehydrated, embedded in paraffin and 5µm slices were acquired for Hematoxylin and Eosin (H&E) staining and 3µm slices were obtained for gram stains. SEM was performed with a Jeol JSM 6610LV (Tokyo, Japan). 16S rRNA Poly chain reaction (PCR) was performed on a CJS sample of both circuits.
